# Delayed Spontaneous Bleeding in the Blind Eye of a Type A Hemophilic Patient

**DOI:** 10.1155/2014/592965

**Published:** 2014-11-09

**Authors:** Gi Sung Son, Sang Un Lee, Sung Chul Kim

**Affiliations:** HanGil Eye Hospital, 543-36 Bupyeong-dong, Bupyeong-gu, Incheon 403-010, Republic of Korea

## Abstract

A 40-year-old man was complaining of severe left ocular pain and headache for the past 2 months. His left eye was traumatized and rendered blind about 20 years ago. He had no other medical problems and his preoperative laboratory tests were nonspecific. Evisceration of the left eye was performed for pain control and cosmetic improvement. However, postoperative progressive and prolonged eyelid swelling, bruising, and wound bleeding recurred. This patient was diagnosed with moderate factor VIII deficiency with a coagulation time within the normal range.

## 1. Introduction

Hemophilia A (factor VIII deficiency) is the most common and serious congenital coagulation disorder. According to the degree of coagulation factor deficiency, bleeding tendencies differ. In the case of moderate deficiency (1–5%), in our patient, spontaneous bleeding is rare [1]. Our patient had not recognized his medical problem before surgery, and his coagulation time in preoperative laboratory tests was within the normal range. We assume that delayed spontaneous bleeding in the traumatized eye about 2 months earlier resulted in ocular pain and headache. Immediately after evisceration was performed, problems relating to abnormal coagulation, such as progressive and prolonged eyelid swelling, bruising, and wound bleeding, were discovered. Moderate factor VIII deficiency was not detected in our usual preoperative laboratory evaluation. To our knowledge, this is the first case report of evisceration in a patient with unrecognized hemophilia.

## 2. Case Report

**Figure 1:**
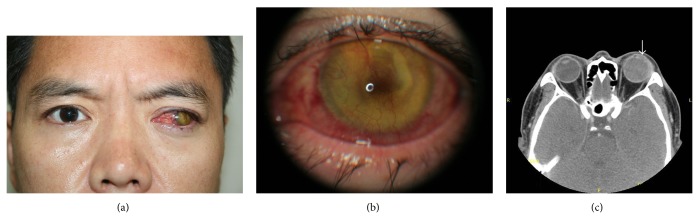
(a) Photograph taken at initial visit showing laterally deviated left eye. Conjunctival injection, corneal opacity, and yellowish pigments behind the cornea are observed. (b) Slit-lamp microscopy examination at initial visit showing corneal neovascularization and an amorphous yellowish lens material with a reddish central pit. (c) Orbital computed tomography scan (axial) at initial visit showing a laterally deviated left eyeball filled with slightly brighter intraocular contents than the contralateral eye. Arrows indicate that the lens material is pushed against the cornea so as to nearly collapse the anterior chamber.

A 40-year-old man presented with left ocular pain that had started 2 months ago. The pain was described as throbbing without diurnal variation. He denied any remarkable events in the past 2 months. His left eye was struck by a piece of wood about 20 years ago, and he had subsequently undergone ocular surgery and was hospitalized for a couple of months. His left eye was blind since the incident, and he has since been using a contact lens for cosmetic purposes. He has no other medical problems. His left eye was laterally deviated with corneal opacity (Figure 1(a)).

Ocular examination with slit-lamp revealed corneal neovascularization with conjunctival injection and yellowish material with a reddish central pit behind the cornea (Figure 1(b)). Best-corrected visual acuity in his left eye was negative for light perception. Intraocular pressure was 39 mmHg as measured by applanation tonometry. Orbital computed tomography (CT) scan showed that his left eye maintained its globe contour and was filled with a homogenous material that was brighter than that of the contralateral eye (Figure 1(c)). The lens seemed to be contracted and pushed against the cornea (Figure 1(c), arrow). Routine preoperative blood work revealed nothing specific, including a coagulation time of 5 min, which is within normal limits (2–6 min).

**Figure 2:**
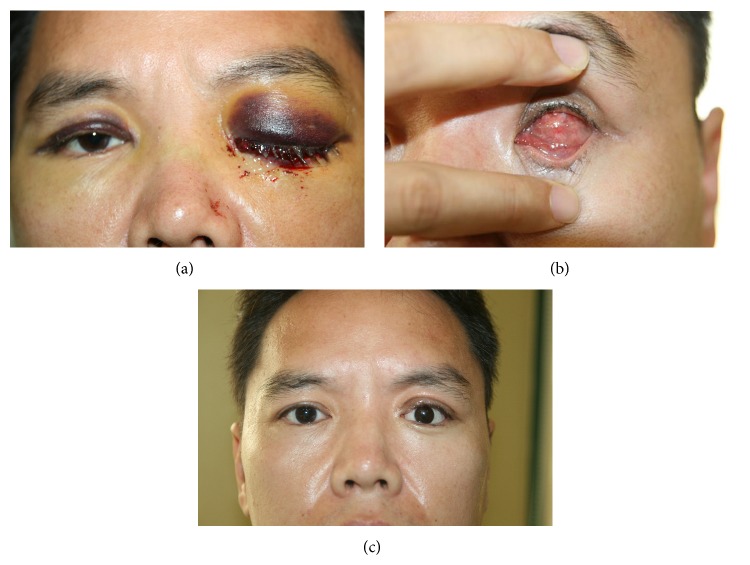
(a) Photograph taken on postoperative day 4. Severe upper eyelid swelling with ecchymosis and conjunctival prolapse through the palpebral fissure due to hemorrhagic chemosis. Note the eyelid ecchymosis extending to the contralateral eye. (b) Photograph taken on postoperative day 13 after infusion of the deficient factor. Good closure of the conjunctiva without chemosis or hemorrhage was observed. (c) Photograph taken on postoperative day 42 showing a well-fitted ocular prosthesis.

Evisceration was performed followed by hydroxyapatite implantation to reduce ocular pain and improve appearance. The procedure was completed unremarkably, but his eyeball was full of mixed fresh and dark blood immediately following corneal buttoning. On the fourth postoperative day, eyelid swelling and prolapsed conjunctiva intensified (Figure 2(a)). The patient was asked to provide more details regarding his history, and he managed to recall a remote bleeding event 10 years ago that had persisted for 15 days after tooth extraction. Prothrombin time/partial thromboplastin time (PT/aPTT) test was performed immediately to assess the patient's bleeding tendency; aPTT was 52 s (reference levels: 27–45 s) and 73 s on the second test, which necessitated quantifying clotting factors. On the fifth postoperative day, compression hemostasis with epinephrine gauze was needed to temporarily stop a considerable amount of bleeding from the conjunctival wound. Postoperative bleeding gradually recurred at shorter intervals until the patient was transfused with factor VIII on the seventh postoperative day. The patient was diagnosed with type A hemophilia with a 4% factor VIII concentration (reference levels: 60–120%). There was no additional bleeding after factor VIII transfusion. The accompanying eyelid swelling and conjunctival prolapse improved on the postoperative day 13 (Figure 2(b)). Individualized ocular prosthesis was created and implanted on the postoperative day 42 (Figure 2(c)).

## 3. Discussion

There are several reports of ophthalmic bleeding events related to hemophilia [2–4]. Furthermore, in these cases, the hemophilic condition was occasionally diagnosed due to unexpected and delayed bleeding after ocular injury or surgery [5, 6]. To date, there is no previous report of undiscovered hemophilia diagnosed after evisceration.

According to coagulation factor deficiency level, bleeding tendencies present differentially. In cases of moderate deficiency (1–5%), as in the present patient, spontaneous bleeding is rare [1]. This fact is the most likely reason why the patient did not recognize his medical problem until the evisceration.

The unidentified hemophilic condition can explain several unusual aspects related to the clinical course in our patient. First, ocular pain began 2 months ago without any remarkable events suggesting delayed spontaneous intraocular bleeding in his traumatized eye. Persistent bleeding without normal hemostasis mechanisms might raise intraocular pressure gradually, which will lead to ocular pain. Second, the volume of mixed fresh and dark blood and the lack of vitreous after corneal buttoning are strongly suggestive of his underlying coagulopathy. Noncoagulated blood could have been confined within the globe cavity. Third, unusual postoperative clinical pictures, including severe eyelid swelling, conjunctival prolapse, and persistent wound bleeding, can be explained by poorly functioning hemostasis.

Our patient's clotting factor VIII concentration was ultimately found to be only 4%. This degree of deficiency falls into the moderate type A hemophilia category [7]. Hemophilia patients in this category are occasionally diagnosed after traumatic events [1].

Missing the hemophilia diagnosis in the present case can be attributed to two reasons. First, detailed history taking was overlooked. Although the patient remembered a prolonged bleeding event after tooth extraction, he was not asked about this until severe eyelid swelling and wound bleeding presented on the fourth postoperative day. Second, routine preoperative tests in our hospital were not sufficiently diverse to detect a mild to moderate hemophilic condition. We consider that coagulation time testing can be positive only in cases of extreme coagulation factor deficiencies and do not consider it a suitable preoperative screening test for mild to moderate type hemophilia.

Appropriate preoperative blood workup is necessary to prevent postoperative complications. First, bleeding time is necessary for checking primary hemostasis function in forming platelet plug at sites of injury. It occurs within seconds of injury. Second, PT for extrinsic and aPTT for intrinsic coagulation pathway are also necessary for checking secondary hemostasis function in forming fibrin. It requires several minutes for completion and prevents recurrent bleeding from occurring hours or days after the initial injury. As mentioned above, ophthalmologists should properly evaluate patient's hemostasis state before every ocular or periocular surgery to prevent unexpected postoperative bleeding.
